# Limited capability of MRI radiomics to predict primary tumor histology of brain metastases in external validation

**DOI:** 10.1093/noajnl/vdae060

**Published:** 2024-04-20

**Authors:** Quirin D Strotzer, Thomas Wagner, Pia Angstwurm, Katharina Hense, Lucca Scheuermeyer, Ekaterina Noeva, Johannes Dinkel, Christian Stroszczynski, Claudia Fellner, Markus J Riemenschneider, Katharina Rosengarth, Tobias Pukrop, Isabel Wiesinger, Christina Wendl, Andreas Schicho

**Affiliations:** Department of Radiology, University Medical Center Regensburg, Regensburg, Germany; Division of Neuroradiology, Department of Radiology, Massachusetts General Hospital, Harvard Medical School, Boston, Massachusetts, USA; Department of Radiology, University Medical Center Regensburg, Regensburg, Germany; Department of Radiology, University Medical Center Regensburg, Regensburg, Germany; Department of Neurosurgery, University Medical Center Regensburg, Regensburg, Germany; Department of Radiology, University Medical Center Regensburg, Regensburg, Germany; Department of Radiology, University Medical Center Regensburg, Regensburg, Germany; Department of Radiology, University Medical Center Regensburg, Regensburg, Germany; Department of Radiology, University Medical Center Regensburg, Regensburg, Germany; Department of Radiology, University Medical Center Regensburg, Regensburg, Germany; Department of Neuropathology, University Medical Center Regensburg, Regensburg, Germany; Department of Neurosurgery, University Medical Center Regensburg, Regensburg, Germany; Department of Internal Medicine III—Hematology and Oncology, University Medical Center Regensburg, Regensburg, Germany; Center of Neuroradiology, medbo District Hospital and University Medical Center Regensburg, Regensburg, Germany; Department of Radiology, University Medical Center Regensburg, Regensburg, Germany; Center of Neuroradiology, medbo District Hospital and University Medical Center Regensburg, Regensburg, Germany; Department of Radiology, University Medical Center Regensburg, Regensburg, Germany

**Keywords:** artificial intelligence, brain metastasis, machine learning, radiomics

## Abstract

**Background:**

Growing research demonstrates the ability to predict histology or genetic information of various malignancies using radiomic features extracted from imaging data. This study aimed to investigate MRI-based radiomics in predicting the primary tumor of brain metastases through internal and external validation, using oversampling techniques to address the class imbalance.

**Methods:**

This IRB-approved retrospective multicenter study included brain metastases from lung cancer, melanoma, breast cancer, colorectal cancer, and a combined heterogenous group of other primary entities (5-class classification). Local data were acquired between 2003 and 2021 from 231 patients (545 metastases). External validation was performed with 82 patients (280 metastases) and 258 patients (809 metastases) from the publicly available Stanford BrainMetShare and the University of California San Francisco Brain Metastases Stereotactic Radiosurgery datasets, respectively. Preprocessing included brain extraction, bias correction, coregistration, intensity normalization, and semi-manual binary tumor segmentation. Two-thousand five hundred and twenty-eight radiomic features were extracted from T1w (± contrast), fluid-attenuated inversion recovery (FLAIR), and wavelet transforms for each sequence (8 decompositions). Random forest classifiers were trained with selected features on original and oversampled data (5-fold cross-validation) and evaluated on internal/external holdout test sets using accuracy, precision, recall, F1 score, and area under the receiver-operating characteristic curve (AUC).

**Results:**

Oversampling did not improve the overall unsatisfactory performance on the internal and external test sets. Incorrect data partitioning (oversampling before train/validation/test split) leads to a massive overestimation of model performance.

**Conclusions:**

Radiomics models’ capability to predict histologic or genomic data from imaging should be critically assessed; external validation is essential.

**Key Points**

- MRI radiomics capability in predicting primary tumor histology of brain metastases is limited.
- Oversampling techniques did not improve classification performance.
- Incorrect data partitioning leads to a massive overestimation of model performance.

**Importance of the Study**

This externally validated study highlights the limitations of radiomics models in predicting the primary tumor histology of brain metastases from MRI. The study emphasizes another significant issue: improper data partitioning can lead to massively inflated assessments of a model’s effectiveness, a problem only discernible through external validation. Methodological errors and a lack of external validation often fuel false hope regarding model performance. Therefore, the methodology of studies that use artificial intelligence should be thoroughly evaluated, and the capability of radiomics models to predict histologic or genomic data from imaging should be critically assessed.

Brain metastases resemble the most common intracranial tumors in adults.^1^ 12.1% of patients with metastasized cancer were found to have brain metastases at diagnosis, frequently being the primary cause of morbidity and mortality.^2^ Often, brain metastases are the initial manifestation of an unknown systemic malignancy.^1,2^ However, targeted therapies are significantly more beneficial than treating cancer of unknown primary.^3^ Therefore, knowledge of primary tumor histology is of utmost importance. This is usually achieved by invasive biopsy, posing the risk of morbidity and mortality.

Artificial intelligence (AI) methods seem suitable for obtaining relevant information from noninvasively acquired imaging data. Few promising models to predict the primary tumor histology from MRI have been presented.^4–6^ Using conventional radiomics, where lesion classification is based on quantitative imaging features, Kniep et al. reported areas under the receiver-operating characteristic curve (AUC) between 0.61 (for breast cancer) and 0.80 (for melanoma) when training only on imaging features. Another recently proposed, sophisticated approach yielded an AUC of 0.88 using a transformer-based deep learning model.^5^ Although promising, no external validation using independent datasets was performed; thereby, the generalizability of the models cannot be assessed, limiting a potential clinical application.

One could argue that suboptimal prediction results are due to the skewed distribution of primary tumors in the datasets used for model development. Imbalanced label distributions pose a major challenge in machine learning applications, particularly in medical domains where minority classes can be critical for accurate diagnosis and treatment. The histological distribution of primary tumor type of patients with brain metastases is highly unbalanced, although exact numbers vary. In a cohort of 729 patients with brain metastases, the most frequent primary tumors found were lung (39%), breast (17%), melanoma (11%), kidney (6%), and gastrointestinal cancer (6%).^7^

Data oversampling techniques, such as the Synthetic Minority Oversampling Technique (SMOTE) have emerged as effective solutions to address this issue.^8^ If applied correctly, the method can improve classification accuracy and reduce bias in datasets with imbalanced label distributions.^9^ However, incorrectly using such methods can lead to overfitting and a dangerous overestimation of model performance.^10^ For example, suppose oversampling is done before the train/test split. In that case, the test set may contain instances that were generated through oversampling techniques and, therefore, seen by the model during training (data leakage).

We aimed to test whether the primary tumor entity can be inferred from MRI-derived radiomic features and whether the results can be maintained on unseen data from an external test set. We further test different oversampling techniques to mitigate class imbalance and show how incorrect data partitioning leads to overestimating classifier performance.

## Materials and Methods

The study followed the Declaration of Helsinki and relevant guidelines and regulations.^11–13^ The institutional review board waived written informed consent and approved this retrospective, multicenter study (approval no. 21-2607-104). The source code is available from https://github.com/qstro/Brainmet-Radiomics.

### Datasets

Local dataset: We included consecutive patients with brain metastases who received oncological care at a university hospital or a tertiary care center between 2003 and 2021. Inclusion criteria were the availability of histological workup and routine MRI with T1w (±contrast agent; T1wCE) and fluid-attenuated inversion recovery (FLAIR) sequences. We only included the first available study after diagnosis of brain metastases and removed patients with incomplete data and segmentations of metastases directly targeted by surgery or stereotactic radiation.

The 2 external datasets consist of the T1w, T1wCE, and FLAIR sequences of a subset of the Stanford University Center for Artificial Intelligence in Medicine and Imaging’s BrainMetShare dataset (*aimi.stanford.edu/brainmetshare;* hereafter: Stanford dataset)^14^ and the Brain Tumor Segmentation (BraTS) Challenge version of the University of California San Francisco Brain Metastases Stereotactic Radiosurgery (UCSF-BMSR) MRI Dataset (*imagingdatasets.ucsf.edu/dataset/1*; hereafter: UCSF dataset).^15^

### Imaging Data Preprocessing

#### Local dataset.

—Images were de-identified and converted to Neuroimaging Informatics Technology Initiative file format (NIFTI) using the dcm2niix tool (v1..20210317; *github.com/rordenlab/dcm2niix*)^16^ and transferred to an on-site Linux Ubuntu (v2.04; Canonical Foundation) workstation. A custom Python routine was implemented for further processing (v3.8.12; Python Software Foundation).

Brain extraction was performed using HD-BET (v1.0; *github.com/MIC-DKFZ/HD-BET*),^17^ removing skull and nonbrain soft tissue. This contributes to anonymization by eliminating the possibility of identifying patients from 3D reconstructions. Volumes were automatically cropped to a bounding box containing the brain. N4 Bias Correction was performed with the SimpleITK N4BiasFieldCorrectionImageFilter (v2.1.0).^18^ All sequences were nonlinearly coregistered to the T1wCE acquisition by rigid, affine, and symmetric diffeomorphic registration using the Advanced Normalization Tools (ANTs; v2.3.5; *stnava.github.io/ANTs*).^19^ Data were subsequently *Z*-score normalized using a brain mask as recommended in the literature.^12,20^

The metastases were automatically voxel-wise segmented into a nonenhancing/necrotic part, an enhancing part, and the surrounding edema using a pretrained nnU-Net.^21^ All segmentations were visually verified and manually corrected using ITK-SNAP (v3.8.0).^22^ Final segmentations were independently approved by 3 board-certified radiologists with 6, 7, and 13 years of neuroradiological experience (A.S., I.W., and C.W.) using a custom-made visual verification tool (QuickSegViewer v1.0; https://github.com/qstro/QuickSegViewer). In case of multiple metastases per patient, segmentations were automatically split into multiple masks using a connected components approach.

#### Stanford dataset.

—Each slice is available as a .png file, with brain extraction and segmentation already performed. Data was converted to NIFTI using the SimpleITK Python library per the acquisition parameters mentioned in the accompanying paper.^14^ The remaining steps (bias correction, coregistration, normalization, and splitting of segmentation masks) were performed as described above.

#### UCSF dataset.

—Provided data are skull-stripped, coregistered, and segmented.^15^ We performed bias correction, normalization, and splitting of the segmentation masks.

### Radiomic Feature Extraction

Before feature extraction, the enhancing and nonenhancing/necrotic tissue segmentations were combined into a single binarized mask representing the tumor core. Radiomic features were extracted separately from each metastasis using the pyradiomics python package (v3.1; *github.com/AIM-Harvard/pyradiomics*) from the FLAIR, T1w, and T1wCE sequences.^23^ Datasets were resampled to an in-plane resolution of 1 mm and a through-plane resolution of 5 mm. The large section thickness results from the different acquisition protocols and our objective of only minimally altering the data using interpolation methods. Fourteen 3D shape-based features were extracted. Furthermore, for each sequence and its wavelet transforms (8 decompositions resulting from applying either a high or a low pass filter in each of the 3 dimensions), 18 first-order statistics, 24 gray level co-occurrences matrix, 16 gray level run length matrix, 16 gray level size zone matrix, 5 neighboring gray-tone difference matrix, and 14 gray level dependence matrix features were extracted.

It has been suggested that the spatial distribution varies between primary tumors.^24^ Therefore, we also included the relative location of each metastasis on the *x*-, *y*-, and *z*-axes as 3 independent features. We computed the metastases’ center of mass and divided it by the shape of the cropped image array, resulting in values between 0 and 1 for each axis.

### Dataset Combinations

We created combinations of the 3 datasets for model training, validation, and testing to assess performance variations due to different study collectives or acquisition protocols. We ensured that the test data always consisted of unseen internal (same study collective) or external data (from other study collectives). The dataset combinations can be obtained from Table 1.

**Table 1.** Dataset Combinations

Combination No.	Training Partition	Testing Partition
1	Local	Stanford + UCSF
2	Stanford	Local + UCSF
3	UCSF	Local + Stanford
4	Local + Stanford	UCSF
5	Local + UCSF	Stanford
6	Stanford + UCSF	Local
7	Local	Stanford
8	Local	USCF
9	Stanford	Local
10	Stanford	UCSF
11	UCSF	Local
12	UCSF	Stanford

Note: All combinations of the included datasets are listed. The training partition is further split into train and validation subsets.

### Data Partitioning

The training partition was split into a train/validation set (80%; *internal train/validation set*) and a holdout test set (20%; *internal test set*) using a label stratified train/test split with nonoverlapping groups as implemented in scikit-learn (v1.0.2).^25^ Data leakage was prevented by assigning all metastases of a patient either to the internal train/validation or the internal test partition.

### Label Selection

Ground truth for the primary tumor entity (*label* or *target variable*) was determined from tissue specimens obtained by open surgery or stereotactic biopsy. Histopathological workup was performed according to general standards using conventional staining methods (H&E and PAS/alcian blue where applicable) and immunohistochemistry.

We selected the 4 most common entities, lung cancer, breast cancer, melanoma, and colorectal cancer and grouped the remaining metastases into a heterogeneous category (*others*) as this classification would be of the largest clinical benefit. In the local dataset, this category includes approximately 20 histological entities, such as kidney and prostate cancer (see Supplementary Figure 1). Metastases with a volume smaller than 125 mm^3^ were removed from further analyses.^4^

### Oversampling

To test different oversampling strategies, we trained a model without oversampling (baseline) and models after applying random oversampling (ROS; randomly duplicating examples in the minority classes) and SMOTE (interjecting datapoints between observations of the minority classes) to the train/validation partition of the internal dataset. Oversampling was done with the scikit-learn-based imbalanced-learn Python library (imblearn v0.11.0; *github.com/scikit-learn-contrib/imbalanced-learn*).^26^ Subsequently, each class had the same number of samples as the most frequent histological entity. Test sets were not oversampled.

#### Incorrect approach.

—To illustrate how incorrect partitioning leads to model overestimation, we trained a model with a slight variation where oversampling occurred before the train/validation/test split. Therefore, the *internal* test set was also oversampled. The *external* test set remained imbalanced.

### Machine Learning Pipeline

As a first step, we tested various combinations of possible modules for the machine learning pipeline using the scikit-learn and imbalanced-learn Python libraries and the train/validation partition of the local dataset.

Since radiomic features should be normalized to avoid feature selection being biased by different orders of magnitude of the variables, we tested *Z*-score normalization (setting the mean of feature values to 0 and the standard deviation to 1) and min–max normalization (scaling all features to lie between 0 and 1).

For machine learning classifiers, reducing the feature space and keeping only relevant features while controlling for the redundancy within these features is essential. This improves model performance, reduces overfitting, and increases interpretability. We tested Maximum Relevance and Minimum Redundancy, Least Absolute Shrinkage and Selection Operator Regression (LASSO), and an ANOVA-based K-Best algorithm for dimensionality reduction and included the 5 best-performing features.

We tested various groups of supervised machine learning classifiers, including linear models (logistic regression), support vector machines (linear and nonlinear), ensemble models (random forest classifier, AdaBoost classifier, and gradient boosting classifier), clustering algorithms (K-nearest neighbors), Bayesian classifiers (Gaussian Naïve Bayes), and neural networks (multilayer perceptron).

In the second step, we used a forward feature selection method to optimize the number of included features using the best-performing pipeline from step 1. To achieve this, we trained multiple iterations of the same pipeline, each time adding the next most important feature as selected by the feature selection algorithm (starting with 1 feature, up to 1% of the initial 2528 features). The cutoff value where the performance stopped increasing was determined as the optimal number of features for the radiomics signature.

To avoid biasing the pipeline toward a single oversampling strategy, we trained versions using no oversampling, ROS, and SMOTE and averaged their results.

### Final Model Training

**Figure 1.**
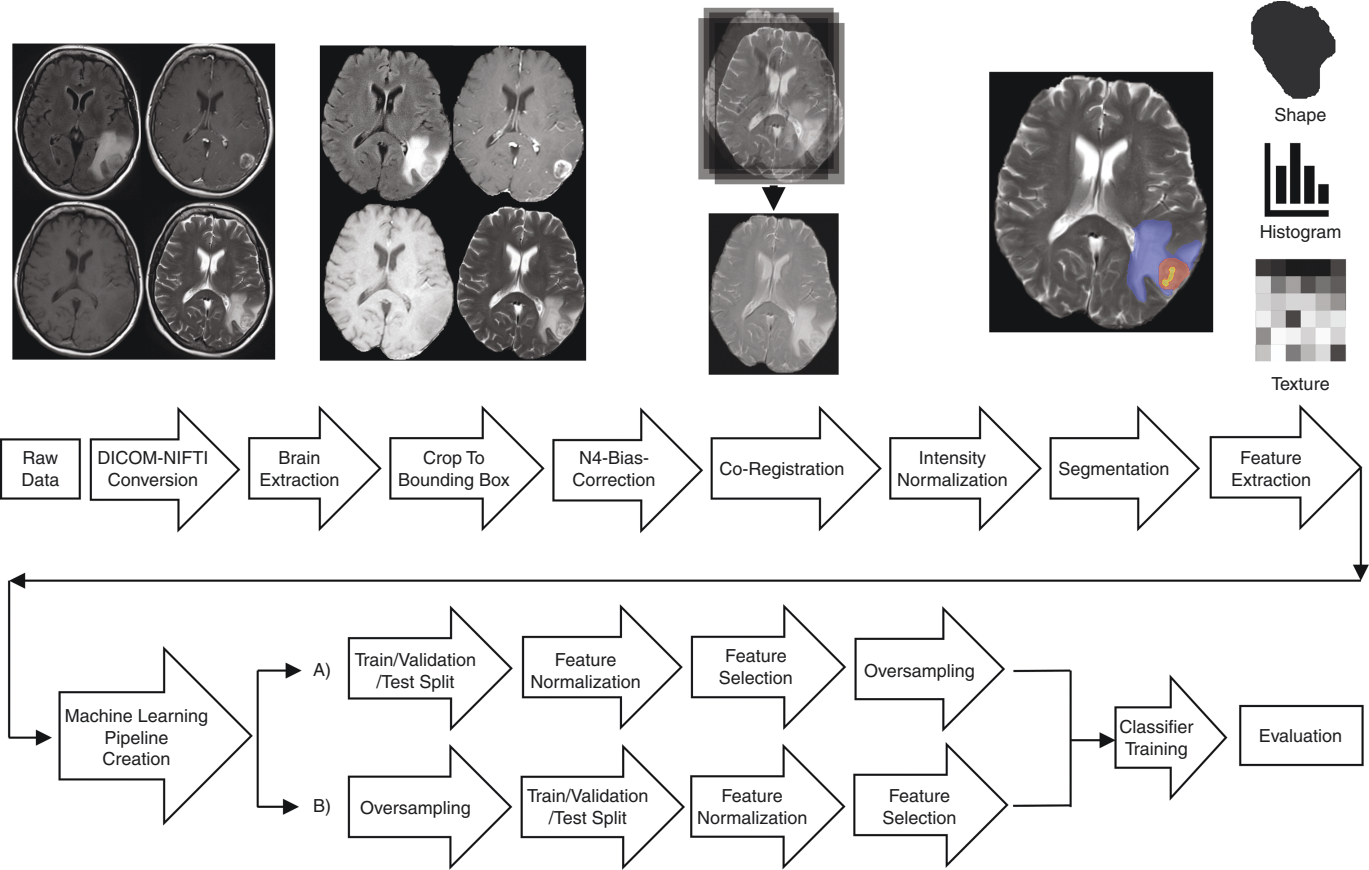
Data processing workflow. The flowchart visualizes the main preprocessing steps. The segmentation depicts the nonenhancing/necrotic part and the enhancing part of the metastasis, as well as the surrounding edema. Note that features were only extracted from the tumor core consisting of enhancing and nonenhancing tumor. (A) *Correct* approach: oversampling performed after partitioning, normalization, and feature selection. (B) *Incorrect* approach: oversampling performed before dataset partitioning.

We finally trained the selected pipeline using the different oversampling strategies based on the internal train/validation partitions of the different dataset combinations for hyperparameter tuning (max features, no. estimators). Feature and label selection models were fitted only with the train/validation partition to prevent data leakage. Normalization was then applied to the train/validation and the internal and external test partitions. The resulting models were assessed with the internal and the external holdout test sets. Figure 1 visualizes all data processing steps.

### Statistical Analysis

Tests were performed using the SciPy library (v1.9.1; *github.com/scipy/scipy*).^27^ Clinical data were compared by descriptive statistics, *t*-tests for continuous data, Mood’s median tests for ordinal data, and Fisher’s exact tests for categorical data. We report continuous data as mean and standard deviation and ordinal data as median and range. Two-tailed tests with a significance level of 0.05 were used. Model evaluation metrics were AUC, accuracy, F1 score, precision, and recall. We used macro-averages of the metrics (averaging the per-class results) as this does not weigh the scores towards the majority class. Ninety-five percent confidence intervals were calculated using a bootstrapping technique with 1000 iterations.

We used a 5-fold cross-validation strategy for all model training steps, optimizing the F1 score for model selection. As the harmonic mean of precision and recall, this metric considers the type of errors the classifier makes, making it robust for evaluating model performance when class distribution is skewed.

We applied a 2-component nonlinear principal component analysis (RBF kernel) to visualize changes to the feature space by different overestimation techniques. Feature importance analysis was conducted to test which radiomic features contribute most to the output. The scikit-learn permutation test score was used to assess whether the models genuinely learned from the data. This function compares the cross-validation score against multiple model versions trained with random permutations (*n* = 100) of the labels, calculating the *P*-value against the null hypothesis that features and targets are independent.

## Results

For 231 patients (111 females) in the local dataset, imaging data (T1w, T1wCE, and FLAIR) were available (primarily 2D acquisitions). Primary tumor histology was available for all patients; however, in 9 cases (10 metastases), it remained a cancer of unknown primary. See Supplementary Figure 1 for the distribution of all primary entities and Supplementary Table 1 for clinical parameters.

Imaging data originated from fifteen different MRI scanner models, including both internally acquired MRI (1.5T Magnetom Avanto, 3.0T Magnetom Skyra, 1.5T Magnetom Symphony, 1.5T Magnetom Aera; all Siemens Healthineers) and external data acquired at 1.0T, 1.5T, and 3.0T scanners. See Supplementary Figures 2 and 3 for scanning details.

**Figure 2.**
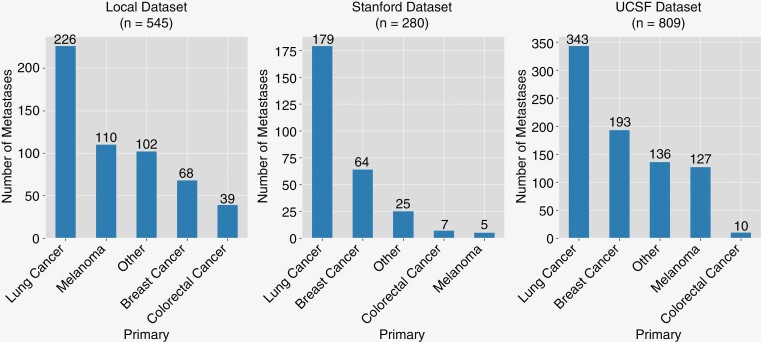
Label Distribution. Number of metastases for each class of the 3 datasets after thresholding at a volume of 125 mm^3^.

Thresholding at a volume of 125 mm^3^ reduced the number of patients (metastases) from 231 (647) to 231 (545) for the local, from 107 (1509) to 82 (280) for the Stanford and from 324 (3349) to 258 (809) for the UCSF datasets. See Figure 2 for the label distribution.

The final model pipeline included *Z*-score normalization, LASSO feature selection with 21 features, and a random forest classifier (advantages include high predictive accuracy, robustness against overfitting, and the capacity to assess feature importance) as this setup yielded the highest F1 scores on the local train-validation set. See Supplementary Tables 2 and 3 for the results of the model selection process.

Nonlinear principal component analysis showed no clear separability of the individual groups in a 2-dimensional setting. The permutation test returned *P*-values < .01 for all model combinations, indicating that all models performed better than chance during cross-validation.

Among all tested dataset combinations, dataset combination 5 (training = Local + UCSF, testing = Stanford) showed the highest F1 score on the external dataset and yielded the most consistent internal and external validation performance (see Supplementary Tables 4 and 5 for detailed results for all dataset combinations). However, even this model did not show convincing classification results with evaluation metrics that were only marginally better than random chance. Table 2 lists the evaluation metrics (macro-averages and per-label) for the baseline model (no oversampling applied) and after ROS and SMOTE (correct and incorrect oversampling approaches). Oversampling did not noticeably improve performance and it did not shift the focus toward the less frequently represented classes.

**Table 2.** Performance Metrics for Dataset Combination 5

		Baseline	SMOTE correct	ROS correct	SMOTE incorrect	ROS incorrect
Number of metastases	Train/validation	1083	2275	2275	2276	2276
	Internal test	271	271	271	569	569
	External test	280	280	280	280	280
	Cross-validation F1 score	0.26	0.81	0.84	0.73	0.81
Random forest parameters	Max features	6	2	2	2	2
	*N* estimators	100	1000	500	500	500
Internal test set	Accuracy	0.38 [0.32,0.44] 0.08; 0;0.77;0.15;0.11	0.37 [0.31,0.42] 0.23;0.1;0.57;0.27;0.19	0.41 [0.35,0.47] 0.21; 0;0.76;0.17;0.13	0.75 [0.72,0.79] 0.75; 1;0.46;0.75,0.8	0.84 [0.81,0.87] 0.82; 1;0.69;0.85;0.83
	F1 score	0.20 [0.16,0.24] 0.11; 0;0.54;0.2;0.15	0.28 [0.22,0.34] 0.27;0.11;0.5;0.29;0.22	0.25 [0.20,0.29] 0.27; 0;0.56;0.22;0.17	0.75 [0.71,0.78] 0.73;0.95;0.53;0.75;0.77	0.84 [0.81,0.87] 0.82;0.98;0.69;0.86;0.84
	Precision	0.24 [0.17,0.31] 0.2; 0;0.42;0.32;0.26	0.29 [0.23,0.37] 0.32;0.12;0.44;0.3;0.26	0.28 [0.22,0.35] 0.38; 0;0.45;0.32;0.27	0.74 [0.71,0.78] 0.7;0.9;0.62;0.75;0.75	0.84 [0.81,0.87] 0.83;0.96;0.69;0.87; 0.84
	Recall	0.22 [0.19,0.25] 0.08; 0;0.77;0.15;0.11	0.27 [0.22,0.34] 0.23;0.1;0.57;0.27;0.19	0.25 [0.22,0.29] 0.21; 0;0.76;0.17;0.13	0.75 [0.72,0.78] 0.75; 1;0.46;0.75;0.8	0.84 [0.81,0.87] 0.82; 1;0.69;0.85;0.83
	AUC	0.59 [0.54,0.65] 0.62;0.57;0.51;0.70;0.53	0.63 [0.57,0.68] 0.64;0.69;0.55;0.66;0.58	0.65 [0.59,0.70] 0.66;0.73;0.54;0.70;0.57	0.93 [0.92,0.95] 0.92; 1;0.87;0.94;0.95	0.97 [0.97,0.98] 0.97;0.1;0.95;0.97;0.98
External test set	Accuracy	0.56 [0.51,0.62] 0.19; 0;0.78;0.60;0.12	0.42 [0.36,0.48] 0.3; 0;0.51;0.80;0.16	0.52 [0.46,0.57] 0.22; 0;0.70;0.80;0.08	0.40 [0.34,0.45] 0.3; 0;0.47;0.6;0.2	0.51 [0.45,0.56] 0.12; 0;0.73;0.2;0.12
	F1 score	0.26 [0.21,0.32] 0.26; 0;0.72;0.15;0.19	0.24 [0.19,0.28] 0.35; 0;0.58;0.10;0.17	0.25 [0.20,0.30] 0.28; 0;0.69;0.15;0.12	0.22 [0.18,0.27] 0.28; 0;0.54;0.13;0.16	0.21 [0.17,0.26] 0.15; 0;0.69;0.07;0.16
	Precision	0.32 [0.23,0.42] 0.43; 0;0.67;0.08;0.43	0.27 [0.22,0.32] 0.42; 0;0.68;0.05;0.19	0.28 [0.21,0.37] 0.38; 0;0.67;0.08;0.29	0.22 [0.19,0.26] 0.27; 0;0.65;0.07;0.14	0.22 [0.17,0.29] 0.19; 0;0.65;0.04;0.23
	Recall	0.34 [0.23,0.43] 0.19; 0;0.78;0.6;0.12	0.35 [0.24,0.42] 0.3; 0;0.51;0.80;0.16	0.36 [0.25,0.43] 0.22; 0;0.70;0.80;0.08	0.31 [0.19,0.42] 0.3; 0;0.47;0.6;0.2	0.24 [0.17,0.34] 0.12; 0;0.73;0.20;0.12
	AUC	0.65 [0.55,0.74] 0.6;0.6;0.55;0.77;0.65	0.62 [0.52,0.71] 0.65;0.42;0.56;0.81;0.63	0.62 [0.54,0.70] 0.63;0.44;0.57;0.81;0.61	0.61 [0.49,0.70] 0.62;0.5;0.57;0.76;0.59	0.61 [0.52,0.69] 0.55;0.48;0.56;0.82;0.58

Note: Performance metrics of the dataset combination with the highest F1 score on the external test set (train/validation = Local + UCSF, test = Stanford). Results for the baseline model (no oversampling) and the correct and incorrect oversampling approaches for SMOTE and ROS are reported. The first line for each performance metric depicts the macro-average with 95% confidence intervals in square brackets. The second line shows the per-label metrics in the order: breast cancer; colorectal cancer; lung cancer; melanoma; and other.

**Figure 3.**
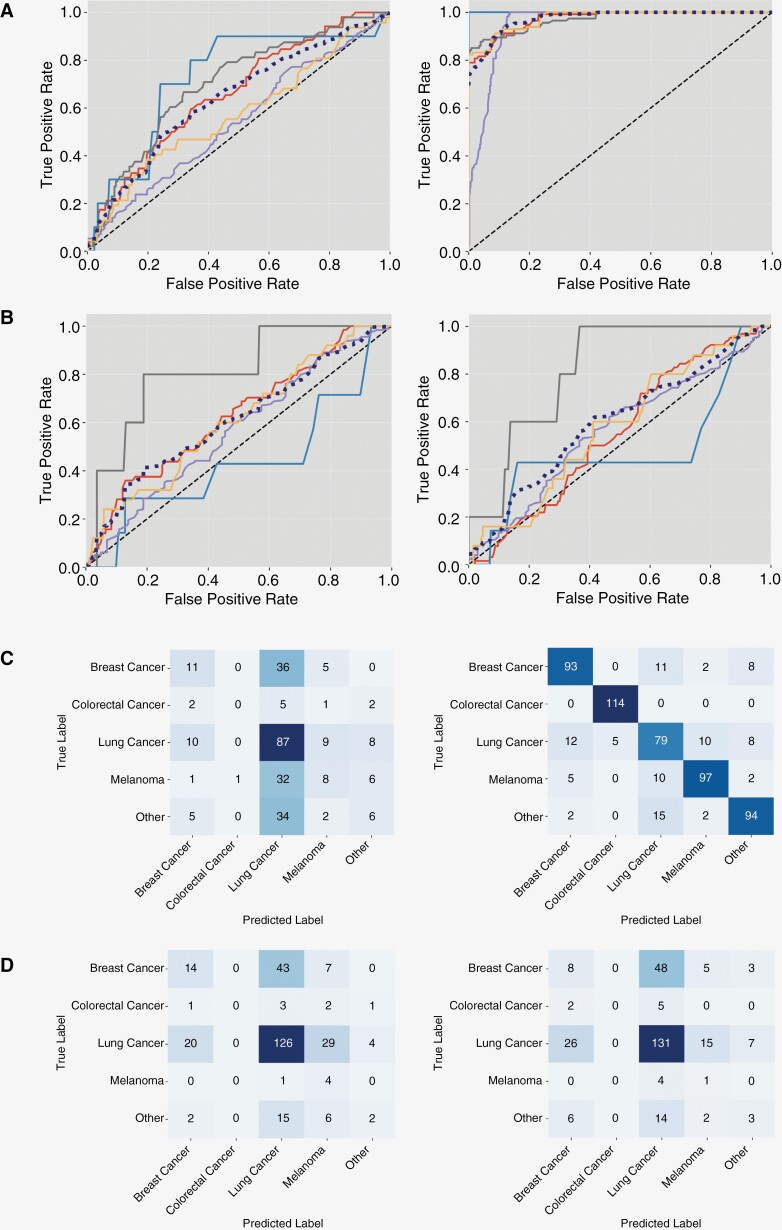
Comparison of correct and incorrect partitioning results. Results for *correct* (left column) and *incorrect* (right column) partitioning results are depicted for dataset combination 5 using the random oversampling technique (train = local + UCSF, test = Stanford). Receiver-operating characteristic curves for the internal (A) and external (B) test sets. Line colors: breast cancer (red), colorectal cancer (light blue), lung cancer (purple), melanoma (gray), other (yellow), and macro-average (dotted dark blue). Random guess (AUC = 0.5) depicted by diagonal dotted lines. The AUC values can be obtained from Table 2. Confusion matrices for internal (C) and external (D) test sets show the number of true and predicted labels from each class.

Differences between correct and incorrect data partitioning are clearly illustrated by the substantial performance differences between internal and external test sets in Figure 3. Here, the false impression of an almost perfect result is given. When looking at the external dataset, it becomes apparent that this performance is not real but the result of an oversampled classifier due to data leakage.

Due to the suboptimal capability to predict the primary entity, the interpretability of feature importance is very limited. The top 5 selected features for dataset combination 5 (baseline model) were all wavelet features and included 2 features extracted from the T1w scans (LLH-firstorder_Maximum, HHL-firstorder_Kurtosis), 2 T1wCE-features (HLH_firstorder_Kurtosis, LHL-firstorder_10Percentile) and 1 FLAIR-feature (LHL-firstorder_RobustMeanAbsoluteDeviation).

## Discussion

Using our local and 2 external datasets, we could not convincingly predict the primary tumor histology of brain metastases using MRI-derived radiomic features in an imbalanced classification task. Oversampling did not improve results and did not shift the focus towards less represented entities. We further showed how incorrect data partitioning can lead to substantial model overestimation.

Several strategies exist to minimize class imbalance. The favored option, obtaining additional examples from minority classes, is not always reasonably achievable, especially in the medical field where the epidemiology of a given disease is a limiting factor. As a solution, generative models are increasingly used to inflate the number of minority-class training cases. For imaging data, generative adversarial networks can synthesize missing MRI sequences. Considering tabular data like extracted radiomic features, ROS and SMOTE are well-established oversampling techniques.

Justified criticism of oversampling techniques exists. For example, it is said that SMOTE hardly affects most classifiers trained on high-dimensional data and is not beneficial for discriminant analysis classifiers, even in the low-dimensional setting.^28^ In our case, oversampling did not improve overall results and made models more prone to overfitting especially when testing classifiers more complex than random forests.

Few studies applying *conventional* radiomics (manually engineered features) exist trying to predict the primaries of brain metastases using MRI-based radiomic features. Ortiz-Ramón et al. report a multiclass AUC of 0.87 in a 3-class task based on 67 brain metastases.^6^ They report unsatisfactory results in distinguishing between breast cancer and melanoma metastases in a one-on-one approach (AUC = 0.61). Comparability to our results is limited, partly because their data came from only 1 scanner, they included only 3 classes, and they chose not to assign metastases from a single patient to either training or test cohorts (potential for data leakage). Our approach more closely resembles that of Kniep et al., who reported AUCs between 0.61 (for breast cancer) and 0.80 (for melanoma) using only imaging features (macro-average AUC,0.69). Their slightly better results may be attributed to the lower heterogeneity of their data. It must be noted that neither study provided results for external test data. Therefore, they do not allow drawing any conclusions regarding generalizability and real-world efficacy.

It is frequently encountered that models performed much worse on external, independent datasets.^29^ This can be due to various reasons, eg differences in the studied collectives or scanning protocols. A non-negligible factor, however, is methodological errors misleading authors into dangerous overestimations of their developed models. *Incorrect* data partitioning, in our case, performing oversampling before dataset splitting, leads to a massive overestimation of the developed classifiers in an overall unsatisfactory classification task. In most cases, however, independent external validation is not performed; thus, these problems remain unnoticed.

A systematic review evaluating AI models for the radiological assessment of COVID-19 demonstrated the widespread prevalence of methodological flaws. The authors concluded that none of the presented models are applicable in clinical practice due to methodological errors and biased training data.^30^ Another review examining the methodological quality of developed AI models from a wide range of disciplines concluded that a significant proportion of developers do not separate train/validation from test sets during preprocessing, leading to data leakage.^31^

Various reporting guidelines have been introduced to ensure correct data processing, machine learning model handling, and the integrity of elementary information in the manuscript. Adherence to these checklists warrants the high quality of the developed models, possible assessment of model generalizability, and reproducibility of the results. Of these tools, a combination of an appropriate scoring system (eg RQS—Radiomics Quality Score or METRICS—METhodological RadiomICs Score)^11,32^ and a dedicated checklist (eg CLEAR—CheckList for EvaluAtion of Radiomics research or CLAIM—Checklist for Artificial Intelligence in Medical Imaging)^33,34^ seems to be suitable for studies like the one presented here as this combination ensures detailed reporting of segmentations, features, data preparation, partitioning, and model architecture. The alignment with appropriate guidelines benefits all stakeholders: authors, reviewers, readers, and most importantly, patients and professional healthcare providers, who can benefit from more reliable models.

With 571 patients (1634 metastases) from 3 independent collectives, our study is the largest to utilize conventional MRI radiomics to predict primary tumor histology of brain metastases (and the only one to be externally validated). It, however, also has several limitations. The multi-scanner, multi-vendor, and multi-site setup could improve generalizability but may decrease cross-validation performance due to inter-scanner and scanning protocol-based variation in radiomic features. Feature robustness could not be tested as per the retrospective nature of the datasets. For the local dataset, we only included the first available study after diagnosis of brain metastases and did not include segmentations directly targeted by radiotherapy or surgery, trying only to include treatment-naïve patients. However, some patients may have already received systemic therapy at the time of the scan. The Stanford dataset is provided in 8-bit*. png* slices (intensity values, 0–255), possibly causing information loss. Also, acquisition parameters between the local (primarily 2D protocols) and external datasets (3D acquisitions) differ considerably and down sampling the through-plane resolution to 5 mm may remove valuable information.

The literature regarding radiomics vs. deep learning approaches is inconclusive. Some applications favor deep learning, namely in breast cancer imaging.^35,36^ Good results in differentiating brain metastases from pathological lung cancer types were found for both approaches.^37^ According to another study, the radiomics approach dominated in differentiating thymic epithelial tumors from other prevascular mediastinal tumors on chest CT.^38^ The preference thus appears to be dependent on the question at hand, which is why we will investigate these different approaches concerning brain metastases in the future.

## Conclusions

Our externally validated study highlights the limitations of MRI-derived radiomics in predicting primary tumor histology of brain metastases. It underscores the critical role of correct study design and external validation, as data leakage can lead to a massive overestimation of model performance. Concerning the unbalanced label distribution, oversampling techniques did not improve classification results. Ultimately, we strongly recommend a comprehensive evaluation of radiomics’ capability to infer histologic or genomic data from imaging studies.

## Supplementary Material

vdae060_suppl_Supplementary_Materials

## Data Availability

Data generated or analyzed during the study are available from the corresponding author by request.
