# Supplementary material for: Limited capability of MRI radiomics to predict primary tumor histology of brain metastases in external validation
Source: Neurooncol Adv. 2024 Apr 20;6(1):vdae060. doi: 10.1093/noajnl/vdae060 (PMC11125388; doi:10.1093/noajnl/vdae060)
Supplement: vdae060_suppl_Supplementary_Materials [file vdae060_suppl_supplementary_materials.docx]

Supplementary Material


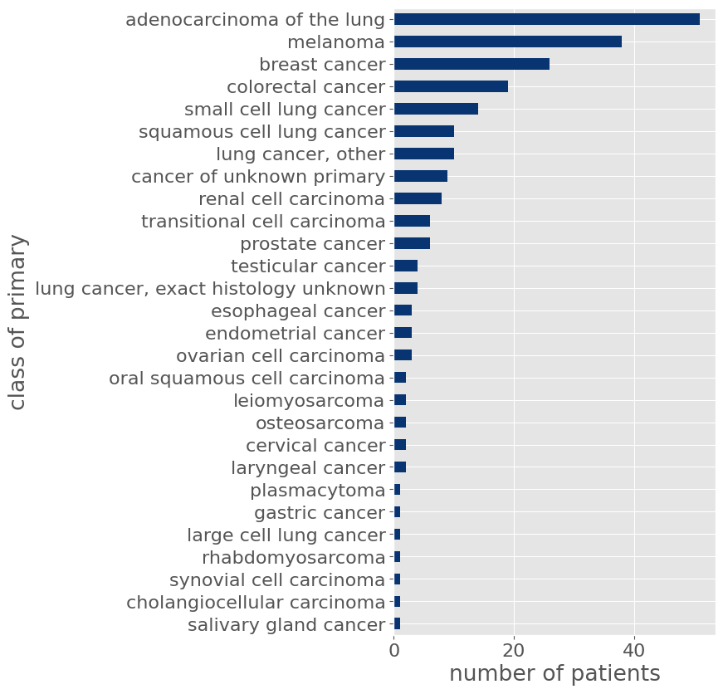

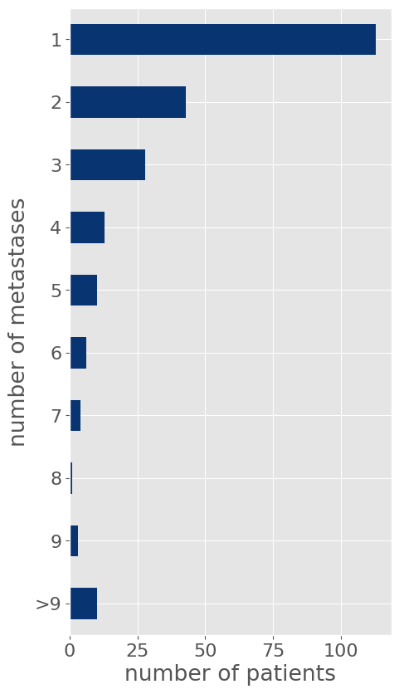


**Supplementary Figure S1: Distribution of Primary Entities and Number of Metastases per Patient (Local Dataset).** Left: label distribution (primary entities) by the number of affected patients. Right: number of metastases per patient. Patients who had more than nine metastases were grouped.


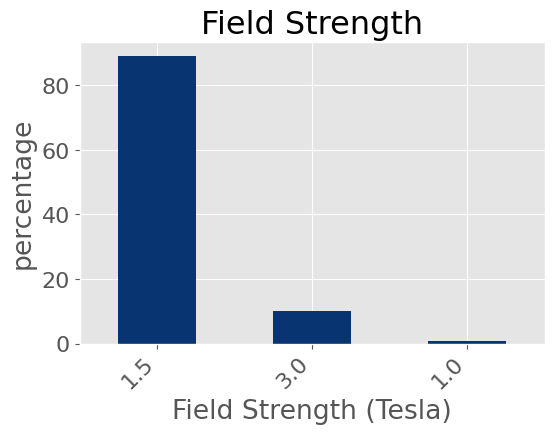

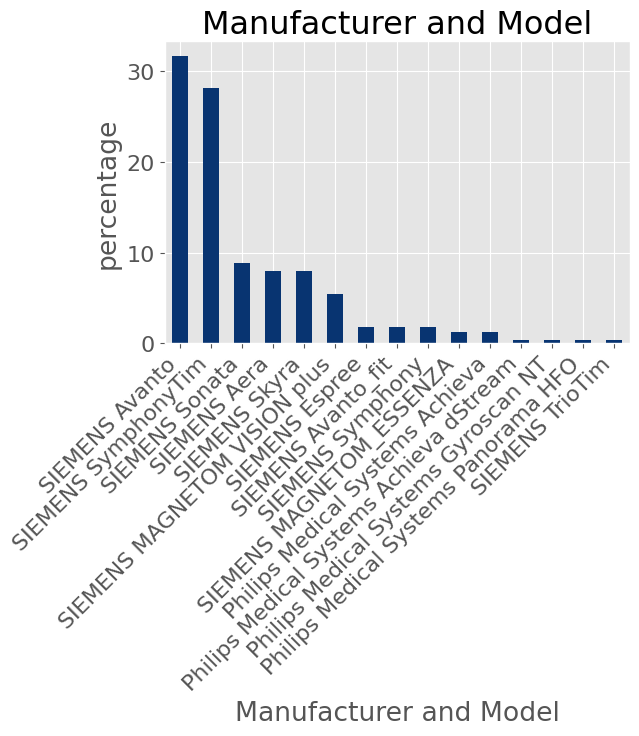


**Supplementary Figure S2:** **MRI Scanner Models and Field Strength (Local Dataset).** Information is taken from DICOM-Tags (0008,0070) – Manufacturer, (0008,1090) – ManufacturerModelName, and (0018,0087) – MagneticFieldStrength.

#
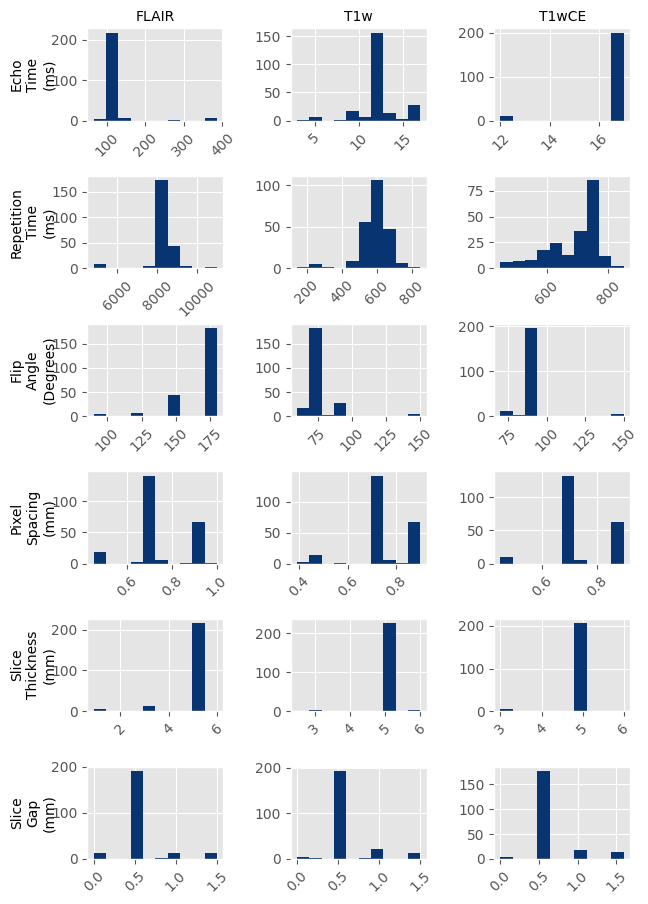


**Supplementary Figure S3: Scanning Details (Local Dataset).** Scanning Details for FLAIR, T1w, and T1wCE Sequences. Information is taken from DICOM-Tags (0018,0081) – EchoTime, (0018,0080) – RepetitionTime, (0018,1314) – FlipAngle, (0028,0030) – PixelSpacing, (0018,0050) – SliceThickness, and slice gap as the difference between slice thickness and (0018,0088) – SpacingBetweenSlices. Units of measurement are shown in parentheses.

#### Supplementary Table S1: Patient Characteristics (Local Dataset)

|  | Number of valid entries | Whole Dataset | Male (*n* = 120) | Female  (*n* = 111) | *P* |
| --- | --- | --- | --- | --- | --- |
| Age (years) | 98.7% (228/231) | 60.47 ± 12.49 | 61.14 ± 13.05 | 59.73 ± 11.85 | .40 |
| Body-Mass-Index (kg/m^2^) | 64.9% (150/231) | 25.66 ± 4.78 | 26.44 ± 4.88 | 24.74 ± 4.54 | .03* |
| Survival (months) | 65.4% (151/231) | 14.34 ± 19.46 | 13.68 ± 19.47 | 15.08 ± 19.56 | .66 |
| ECOG status | 68.8% (159/231) | 1 (0, 4) | 1 (0, 4) | 1 (0, 4) | .61 |
| *Therapy* |  |  |  |  |  |
| Surgery | 97.8% (226/231) | 98.2% (222/226) | 97.5% (115/118) | 99.1% (107/108) | .62 |
| Complete Resection | 88.7% (205/231) | 32.2% (66/205) | 32.4% (35/108) | 32.0% (31/97) | 1.0 |
| Radiation Therapy | 98.7% (228/231) | 64.9% (148/228) | 66.7% (80/120) | 63.0% (68/108) | .58 |
| Chemotherapy | 97.4% (225/231) | 39.1% (88/225) | 41.0% (48/117) | 37.0% (40/108) | .59 |
| Immunotherapy | 97.4% (225/231) | 12.9% (29/225) | 12.8% (15/117) | 13.0% (14/108) | 1.0 |
| *Further Organ Metastases* |  |  |  |  |  |
| Hepatic | 98.7% (228/231) | 19.7% (45/228) | 16.7% (20/120) | 23.1% (25/108) | .25 |
| Pulmonary | 98.7% (228/231) | 37.3% (85/228) | 38.3% (46/120) | 36.1% (39/108) | .78 |
| Osseous | 98.7% (228/231) | 21.5% (49/228) | 23.3% (28/120) | 19.4% (21/108) | .52 |
| Lymphogenic | 98.7% (228/231) | 32.5% (74/228) | 36.7% (44/120) | 27.8% (30/108) | .16 |
| Adrenal | 98.7% (228/231) | 11.0% (25/228) | 12.5% (15/120) | 9.3% (10/108) | .53 |

Note.—Characteristics of the whole dataset and separated by sex. Numbers are reported as mean and standard deviation for continuous data, median and range for ordinal data (ECOG performance status), and percentage for categorical data. Survival is calculated from the first diagnosis of brain metastases until death. Missing survival values correspond to cases where the date of death is unknown or patients are still alive. *P* values correspond to the statistical comparison between sex. *significant by the alpha level of .05.

#### Supplementary Table S2: Best Performing Module Combinations for Classifier Selection

| Rank | Feature Normalization Technique | Feature Selection Method | Oversampling Technique | Machine Learning Classifier | Accuracy | AUC | F1 |
| --- | --- | --- | --- | --- | --- | --- | --- |
| 1 | **Z-Score** | **LASSO** | **Random** | **Random Forest** | **.313** | **.563** | **.243** |
| 2 | **Z-Score** | **LASSO** | **SMOTE** | **Random Forest** | **.304** | **.552** | **.242** |
| 3 | Z-Score | LASSO | None | K-Nearest Neighbors | .355 | .559 | .237 |
| 4 | Min-Max | K-Best | None | K-Nearest Neighbors | .367 | .531 | .235 |
| 5 | Z-Score | K-Best | SMOTE | Gradient Boosting | .277 | .534 | .234 |
| 6 | Z-Score | LASSO | SMOTE | Gradient Boosting | .285 | .536 | .233 |
| 7 | Min-Max | LASSO | None | Gradient Boosting | .372 | .548 | .23 |
| 8 | Z-Score | LASSO | SMOTE | AdaBoost | .254 | .532 | .229 |
| 9 | Z-Score | MRMR | None | Non-Linear Support Vector Machine | .263 | .526 | .228 |
| 10 | Min-Max | K-Best | None | Gradient Boosting | .344 | .557 | .225 |
| 11 | **Z-Score** | **LASSO** | **None** | **Random Forest** | **.359** | **.558** | **.222** |
| 12 | Z-Score | K-Best | None | Random Forest | .358 | .552 | .222 |
| 13 | Min-Max | K-Best | None | Random Forest | .366 | .552 | .221 |
| 14 | Min-Max | K-Best | Random | Random Forest | .312 | .529 | .221 |
| 15 | Z-Score | LASSO | SMOTE | Non-Linear Support Vector Machine | .285 | .559 | .221 |
| 16 | Z-Score | LASSO | None | Multilayer Perceptron | .403 | .558 | .22 |
| 17 | Z-Score | K-Best | None | K-Nearest Neighbors | .355 | .533 | .22 |
| 18 | Min-Max | LASSO | SMOTE | Random Forest | .302 | .546 | .22 |
| 19 | Z-Score | LASSO | Random | K-Nearest Neighbors | .239 | .529 | .219 |
| 20 | Z-Score | K-Best | SMOTE | Multilayer Perceptron | .223 | .596 | .219 |
| 21 | Z-Score | K-Best | Random | Random Forest | .307 | .535 | .218 |
| 22 | Z-Score | LASSO | SMOTE | Support Vector Machine | .239 | .568 | .218 |
| 23 | Z-Score | K-Best | None | Gradient Boosting | .337 | .555 | .217 |
| 24 | Min-Max | LASSO | SMOTE | Gradient Boosting | .278 | .545 | .217 |
| 25 | Z-Score | K-Best | Random | Multilayer Perceptron | .225 | .597 | .216 |

Note.—A multitude of possible combinations of feature normalization, feature selection, and machine learning classifiers were tested on the local train/validation data (stratified five-fold cross-validation). Each combination was trained with and without oversampling of the training partition. Only the 25 best-performing combinations are listed, sorted by F1-score. The combination with the best aggregate F1-score for all three oversampling techniques was selected for the machine learning pipeline (highlighted in bold letters).

**Supplementary Table S3: Selected Number of Features**

| Rank | Number of Features | Oversampling Technique | Accuracy | AUC | F1-Score |
| --- | --- | --- | --- | --- | --- |
| 1 | **24** | **SMOTE** | **.38** | **.608** | **.291** |
| 2 | 23 | SMOTE | .375 | .606 | .289 |
| 3 | 25 | SMOTE | .362 | .596 | .284 |
| 4 | 21 | SMOTE | .366 | .587 | .282 |
| 5 | **18** | **Random** | **.392** | **.585** | **.28** |
| 6 | 18 | SMOTE | .352 | .593 | .279 |
| 7 | 19 | SMOTE | .373 | .587 | .277 |
| 8 | 20 | Random | .394 | .576 | .271 |
| 9 | 17 | Random | .385 | .573 | .271 |
| 10 | 22 | SMOTE | .359 | .588 | .269 |
| 11 | 24 | Random | .403 | .609 | .268 |
| 12 | 25 | Random | .394 | .583 | .268 |
| 13 | 19 | Random | .384 | .591 | .265 |
| 14 | 16 | Random | .378 | .588 | .265 |
| 15 | 16 | SMOTE | .343 | .575 | .262 |
| 16 | 20 | SMOTE | .352 | .592 | .261 |
| 17 | 6 | Random | .361 | .541 | .26 |
| 18 | 15 | SMOTE | .347 | .579 | .255 |
| 19 | 21 | Random | .376 | .588 | .253 |
| 20 | 14 | Random | .362 | .582 | .25 |
| 21 | 17 | SMOTE | .328 | .575 | .249 |
| 22 | **22** | **None** | **.419** | **.602** | **.248** |
| 23 | 11 | SMOTE | .316 | .56 | .248 |
| 24 | 5 | SMOTE | .297 | .556 | .247 |
| 25 | 14 | SMOTE | .339 | .587 | .246 |

Note.— Results for determining cutoff feature number (dimensionality reduction) using a forward feature selection method. Multiple iterations of the same pipeline were evaluated, starting with the most important feature and adding the next most important feature (as determined by lasso regression) with each iteration (up to 1% of the initial 2528 features). The final number of features was determined by averaging the best-performing feature numbers for the three oversampling techniques.

**Supplementary Table S4: Performance Metrics for Dataset Combinations 1-6**

|  |  | Baseline | | | | | | SMOTE | | | | | | Random Oversampling | | | | | |
| --- | --- | --- | --- | --- | --- | --- | --- | --- | --- | --- | --- | --- | --- | --- | --- | --- | --- | --- | --- |
| Dataset Comb. | No. | 1 | 2 | 3 | 4 | 5 | 6 | 1 | 2 | 3 | 4 | 5 | 6 | 1 | 2 | 3 | 4 | 5 | 6 |
|  | Train/Val./Int. Test | Loc. | SU | UCSF | Loc.  +SU | Loc.+  UCSF | UCSF  +SU | Loc. | SU | UCSF | Loc.  +SU | Loc.+  UCSF | UCSF  +SU | Loc. | SU | UCSF | Loc.  +SU | Loc.+  UCSF | UCSF  +SU |
|  | External Test | SU+  UCSF | Loc.+  UCSF | Loc.  +SU | UCSF | SU | Loc. | SU+  UCSF | Loc.+  UCSF | Loc.  +SU | UCSF | SU | Loc. | SU+  UCSF | Loc.+  UCSF | Loc.  +SU | UCSF | SU | Loc. |
| Number of Metastases | Train/Validation | 437 | 227 | 646 | 660 | 1083 | 871 | 905 | 715 | 1370 | 1620 | 2275 | 2085 | 905 | 715 | 1370 | 1620 | 2275 | 2085 |
|  | Internal Test | 108 | 53 | 163 | 165 | 271 | 218 | 108 | 53 | 163 | 165 | 271 | 218 | 108 | 53 | 163 | 165 | 271 | 218 |
|  | External Test | 1089 | 1354 | 825 | 809 | 280 | 545 | 1089 | 1354 | 825 | 809 | 280 | 545 | 1089 | 1354 | 825 | 809 | 280 | 545 |
|  | Cross-Val. F1 score | .27 | .31 | .33 | .23 | .26 | .3 | .81 | .94 | .84 | .85 | .81 | .84 | .87 | .97 | .87 | .92 | .84 | .89 |
| Random Forest Parameters | Max features | 6 | 6 | 6 | 2 | 6 | 6 | 2 | 2 | 2 | 2 | 2 | 2 | 2 | 2 | 2 | 2 | 2 | 2 |
|  | N estimators | 100 | 10 | 1000 | 10 | 100 | 10 | 500 | 500 | 1000 | 1000 | 1000 | 1000 | 500 | 1000 | 500 | 1000 | 500 | 1000 |
| Internal Test Set | Accuracy | .29 | .68 | .42 | .44 | .38 | .46 | .31 | .68 | .39 | .44 | .37 | .43 | .32 | **.72** | .43 | .48 | .41 | .49 |
|  | F1 Score | .13 | .3 | .25 | .19 | .2 | .28 | .23 | **.42** | .28 | .3 | .28 | .29 | .21 | .41 | .26 | .22 | .25 | .28 |
|  | Precision | .12 | .32 | .31 | .2 | .24 | .3 | .23 | **.57** | .28 | .32 | .29 | .29 | .35 | .46 | .28 | .28 | .28 | .35 |
|  | Recall | .16 | .3 | .25 | .21 | .22 | .28 | .23 | .39 | .28 | .3 | .27 | .29 | .21 | **.42** | .27 | .24 | .25 | .28 |
|  | AUC | .54 | .59 | .66 | .5 | .59 | .59 | .64 | **.75** | .66 | .62 | .63 | .61 | .58 | .7 | .64 | .6 | .65 | .6 |
| External Test Set | Accuracy | .37 | .41 | .43 | .36 | **.56** | .34 | .38 | .4 | .4 | .37 | .42 | .34 | .42 | .42 | .44 | .41 | .52 | .39 |
|  | F1 Score | .22 | .17 | .2 | .19 | **.26** | .17 | .22 | .17 | .25 | .23 | .24 | .15 | .24 | .15 | .23 | .18 | .25 | .13 |
|  | Precision | .22 | .21 | .22 | .22 | .32 | .22 | .23 | .25 | .23 | .27 | .27 | .16 | .25 | **.35** | .25 | .26 | .28 | .31 |
|  | Recall | .22 | .22 | .22 | .22 | .34 | .21 | .23 | .22 | .27 | .24 | .35 | .18 | .24 | .21 | .25 | .21 | **.36** | .19 |
|  | AUC | .53 | .52 | .57 | .52 | **.65** | .49 | .57 | .55 | .56 | .58 | .62 | .51 | .55 | .54 | .57 | .6 | .62 | .52 |

Note.—Number of metastases in train/validation/test partitions and evaluation metrics for internal and external test sets are reported for each dataset combination and oversampling technique (baseline = no oversampling performed). The best evaluation results for each row are highlighted in bold. Abbreviations: Loc. = local dataset, SMOTE = Synthetic Minority Oversampling Technique, SU = Stanford University, UCSF = University of California San Francisco.

**Supplementary Table S5: Performance Metrics for Dataset Combinations 7-12**

|  |  | Baseline | | | | | | SMOTE | | | | | | Random Oversampling | | | | | |
| --- | --- | --- | --- | --- | --- | --- | --- | --- | --- | --- | --- | --- | --- | --- | --- | --- | --- | --- | --- |
| Dataset Combination | No. | 7 | 8 | 9 | 10 | 11 | 12 | 7 | 8 | 9 | 10 | 11 | 12 | 7 | 8 | 9 | 10 | 11 | 12 |
|  | Train/Val./Int. Test | Local | Local | SU | SU | UCSF | UCSF | Local | Local | SU | SU | UCSF | UCSF | Local | Local | SU | SU | UCSF | UCSF |
|  | External Test | SU | UCSF | Local | UCSF | Local | SU | SU | UCSF | Local | UCSF | Local | SU | SU | UCSF | Local | UCSF | Local | SU |
| Number of Metastases | Train/Validation | 437 | 437 | 227 | 227 | 646 | 646 | 905 | 905 | 715 | 715 | 1370 | 1370 | 905 | 905 | 715 | 715 | 1370 | 1370 |
|  | Internal Test | 108 | 108 | 53 | 53 | 163 | 163 | 108 | 108 | 53 | 53 | 163 | 163 | 108 | 108 | 53 | 53 | 163 | 163 |
|  | External Test | 280 | 809 | 545 | 809 | 545 | 280 | 280 | 809 | 545 | 809 | 545 | 280 | 280 | 809 | 545 | 809 | 545 | 280 |
|  | Cross-Validation F1 score | .27 | .27 | .31 | .31 | .33 | .33 | .81 | .81 | .94 | .94 | .84 | .84 | .87 | .87 | .97 | .97 | .87 | .87 |
| Random Forest Parameters | Max features | 6 | 6 | 6 | 6 | 6 | 6 | 2 | 2 | 2 | 2 | 2 | 2 | 2 | 2 | 2 | 2 | 2 | 2 |
|  | N estimators | 100 | 100 | 10 | 10 | 1000 | 1000 | 500 | 500 | 500 | 500 | 1000 | 1000 | 500 | 500 | 1000 | 1000 | 500 | 500 |
| Internal Test Set | Accuracy | .29 | .29 | .68 | .68 | .42 | .42 | .31 | .31 | .68 | .68 | .39 | .39 | .32 | .32 | **.72** | **.72** | .43 | .43 |
|  | F1 Score | .13 | .13 | .3 | .3 | .25 | .25 | .23 | .23 | **.42** | **.42** | .28 | .28 | .21 | .21 | .41 | .41 | .26 | .26 |
|  | Precision | .12 | .12 | .32 | .32 | .31 | .31 | .23 | .23 | **.57** | **.57** | .28 | .28 | .35 | .35 | .46 | .46 | .28 | .28 |
|  | Recall | .16 | .16 | .3 | .3 | .25 | .25 | .23 | .23 | .39 | .39 | .28 | .28 | .21 | .21 | **.42** | **.42** | .27 | .27 |
|  | AUC | .54 | .54 | .59 | .59 | .66 | .66 | .64 | .64 | **.75** | **.75** | .66 | .66 | .58 | .58 | .7 | .7 | .64 | .64 |
| External Test Set | Accuracy | .45 | .34 | .4 | .41 | .36 | .56 | .44 | .36 | .4 | .4 | .35 | .52 | .49 | .39 | .42 | .42 | .36 | **.59** |
|  | F1 Score | .2 | .21 | .12 | .18 | .16 | .26 | .21 | .21 | .14 | .18 | .22 | **.27** | .2 | .24 | .13 | .16 | .19 | **.27** |
|  | Precision | .2 | .21 | .13 | .23 | .19 | .29 | .24 | .23 | .23 | .25 | .2 | .28 | .2 | .26 | **.35** | **.35** | .21 | .29 |
|  | Recall | .22 | .22 | .19 | .22 | .2 | .27 | .23 | .22 | .2 | .22 | .25 | **.36** | .26 | .25 | .2 | .21 | .21 | .29 |
|  | AUC | .53 | .55 | .5 | .54 | .54 | .56 | .57 | .59 | .54 | .51 | .52 | **.6** | .58 | .58 | .53 | .5 | .53 | **.6** |

Note.—Number of metastases in train/validation/test partitions and evaluation metrics for internal and external test sets are reported for each dataset combination and oversampling technique (baseline = no oversampling performed). The best evaluation results for each row are highlighted in bold. Abbreviations: SMOTE = Synthetic Minority Oversampling Technique, SU = Stanford University, UCSF = University of California San Francisco.
